# The Impact of Gadolinium on Quantitative Myelin Metrics in Ex Vivo Spinal Cord

**DOI:** 10.1002/nbm.70275

**Published:** 2026-03-22

**Authors:** Hannah E. Alderson, Mark D. Does, Kevin D. Harkins

**Affiliations:** ^1^ Department of Biomedical Engineering Vanderbilt University Nashville Tennessee USA; ^2^ Vanderbilt University Institute of Imaging Science Nashville Tennessee USA; ^3^ Department of Electrical Engineering Vanderbilt University Nashville Tennessee USA; ^4^ Department of Radiology and Radiological Sciences Vanderbilt University Medical Center Nashville Tennessee USA

**Keywords:** gadolinium, myelin, quantitative MRI, spinal cord

## Abstract

The goal of this work was to evaluate the impact of gadolinium on quantitative myelin MRI metrics in ex vivo tissue. Ex vivo ferret spinal cords were imaged with and without gadolinium. Mean metrics were calculated across white and gray matter. T_1_ of the free proton pool and T_2_ of water other than myelin showed the largest differences between gadolinium and without gadolinium samples. Myelin water fraction, bound pool fraction, and myelin water T_2_ showed a 2%–12% change between gadolinium and no gadolinium data. The relationships between the myelin water fraction, bound pool fraction, and myelin water T_2_ and a recently established axon diameter surrogate were not affected by gadolinium; however, the trends observed between T_1_ of the free proton pool and other water T_2_ and the axon diameter surrogate without gadolinium were flattened in the samples with gadolinium. Overall, the impacts of gadolinium were primarily observed in the relaxation parameters of the non‐myelin proton pools.

## Graphical abstract

Quantitative MRI myelin metrics were evaluated in ex vivo ferret spinal cord with and without gadolinium (Gd). T_1_ of the free proton pool (T_1f_) and T_2_ of the intracellular and extracellular spaces (OWT_2_) were both significantly shorter with Gd, as expected. Metrics reporting on myelin content show significant differences between Gd and no‐Gd datasets when averaged across the white matter; however, the magnitude of these differences are likely not of concern in many applications.

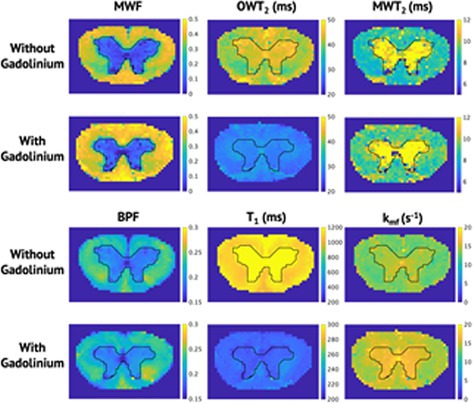

**Abbreviations**
∆D_⊥_
: difference in radial diffusivity with diffusion time
BPF: bound pool fraction
D_⊥_
: radial diffusivity
DW‐MRI: diffusion‐weighted MRI
FOV: field of view
Gd: gadolinium
MET_2_
: multi‐exponential T_2_
MSE: multiple spin echo
MWF: myelin water fraction
MWT_2_
: myelin water T_2_
NEX: number of excitations
OGSE: oscillating spin echo
OWT_2_
: other water T_2_
PGSE: pulsed gradient spin echo
PSR: pool size ratio
qMRI: quantitative MRI
qMT: quantitative magnetization transfer
R_1_
: longitudinal relaxation rate
R_2_
: transverse relaxation rate
RARE: rapid acquisition relaxation enhancement
ROIs: region of interests
SIR: selective inversion recovery
T_1f_
: T_1_ of the free proton pool
TI: inversion times
TR: repetition time

## 1 Introduction

Gadolinium (Gd) is a well‐characterized contrast agent in MRI, known to increase both longitudinal (R_1_) [1, 2, 3] and transverse (R_2_) [4, 5] relaxation rates. While Gd provides diagnostic benefits by enhancing contrast between healthy and diseased tissue (e.g., tumors) for in vivo applications, it is used most commonly in ex vivo tissue samples to reduce the experimental repetition time (TR) and ultimately scan times—an important benefit, particularly in the case of high‐resolution diffusion‐weighted MRI (DW‐MRI) [5, 6] where scans can be several hours long.

Gd is chelated to reduce its toxicity, limiting its ability to bind to other molecules and permeate cell membranes [7]. Therefore, Gd infused for in vivo applications causes signal enhancement only in tissues where the chelate is able to permeate the vasculature, including regions where the blood brain barrier is compromised [7, 8]. Its distribution in ex vivo tissues that have been emersed in Gd solution is less clear. It is often assumed that Gd is present everywhere there is water; however, some studies in mouse brains were interpreted to indicate that there may also be compartments that are inaccessible to Gd [4]. Additionally, work published by Stanisz and Henkelman found the relaxivity of Gd increased with increased macromolecular content [9].

Any unequal distribution of Gd could impact quantitative results for ex vivo MRI studies that use Gd to shorten scan time. Quantitative magnetic resonance imaging (qMRI) metrics—such as quantitative magnetization transfer (qMT) analysis of selective inversion recovery (SIR) data and multi‐exponential T_2_ (MET_2_) analysis of multiple spin echo (MSE)—rely on assumptions about relaxation in different compartments. For instance, myelin water fraction (MWF) from MET_2_ analysis assumes that compartments can be distinguished by T_2_ relaxation rates and that signal fractions from myelin water and non‐myelin water compartments experience slow exchange [10, 11, 12]. Similarly, pool size ratio (PSR) from qMT analysis depends on assumptions about T_1_ relaxation rates caused by exchange between free and macromolecular pools of protons [11, 13]. Both methods serve to quantify myelin content and are commonly used in ex vivo investigations.

This work presents preliminary findings on the impact of Gd to shorten scan time on quantitative relaxometry techniques for ex vivo MRI in a small number of samples (*N* = 3). The results compare MET_2_ and qMT metrics in ex vivo ferret spinal cords with and without Gd. Lastly, the relationship of these metrics to a recently validated diffusion MRI‐derived axon diameter surrogate, ∆D_⊥_, is also evaluated with and without Gd.

## 2 Methods

### 2.1 Tissue Preparation

All animal procedures were completed in compliance with the Vanderbilt University Institutional Animal Care and Use Committee. Methods of tissue extraction and fixation are as described in Alderson et al. [14]. Briefly, adult male ferrets (*N* = 3) were anesthetized, and a transcardial perfusion was performed [15]. Spinal cords were fixed post‐extraction for 10 days and then rehydrated and stored long term initially in 1× PBS, 1 mM Gd (ProHance), and 0.01% sodium azide. Following MRI with Gd, tissues were washed every 2 to 3 days for a total of 14 days with 1× PBS and 0.01% sodium azide to clear Gd from the spinal cords. The present work is motivated by prior experiments [14], where the three spinal cords from that work that were not used for histology were re‐scanned with and without Gd.

### 2.2 MRI Acquisitions

All MRI acquisitions were completed on a 15.2‐T Bruker Biospec Avance III horizontal scanner (Billerica, MA).

#### 2.2.1 Acquisitions With Gd

MSE measurements were acquired with the first echo time = 6.5 ms, echo spacing = 6.5 ms, number of echoes = 20, TR = 600 ms, and number of excitations (NEX) = 30. SIR measurements were also made with 12 inversion times (TI) log‐spaced from 10 to 1500 ms, delay time = 600 ms, NEX = 6, and rapid acquisition relaxation enhancement (RARE) factor = 4. Lastly, two diffusion‐weighted imaging acquisitions were completed, one with an oscillating spin echo (OGSE) sequence (${∆}_{\mathrm{eff}}$ = 2.5 ms, cosine with duration = 10 ms, and number of periods = 1) and one with a pulsed gradient spin echo (PGSE) sequence (${∆}_{\mathrm{eff}}$ = 25 ms, ∆ = 26 ms, and *δ* = 3 ms). For each diffusion‐weighted acquisition, echo time = 40 ms, *b* = 800 s/mm^2^, 1 *b* = 0 image, 15 diffusion encoded images, and NEX = 4. Total acquisition time for three spinal cords with Gd was 15.5 h.

#### 2.2.2 Acquisitions Without Gd

MSE measurements were modified to have a TR = 4500 ms and NEX = 12. SIR acquisitions were also modified with 12 TIs log‐spaced from 10 to 9000 ms. Diffusion‐weighted imaging was performed with TR = 4500 ms and NEX = 6.

For all acquisitions, three spinal cords were imaged at one time with a field of view (FOV) of 15 × 15 mm, matrix 120 × 120, 125 μm^2^ in‐plane resolution, and 2 mm slice thickness. Total acquisition time for three spinal cords without Gd was 38 h.

### 2.3 Data Analysis

Reconstruction and analyses were completed in MATLAB R2024b (The Mathworks, Natick, MA). MET_2_, qMT, and diffusion tensor analyses were performed on a voxel‐wise basis using the REMMI toolbox as described in Alderson et al. [14]. MET_2_ analysis allows for the estimation of myelin water T_2_ (MWT_2_), intracellular and extracellular or “other” water T_2_ (OWT_2_), and MWF. qMT analysis of SIR data allows for the estimation of T_1_ of the free proton pool (T_1f_) and bound pool fraction (BPF). Finally, standard diffusion tensor analysis provides radial diffusivity (D_⊥_), and ∆D_⊥_ is calculated by subtracting D_⊥_ measured via PGSE at the long diffusion time from D_⊥_ measured via OGSE at the short diffusion time [14, 16].

**FIGURE 1:**
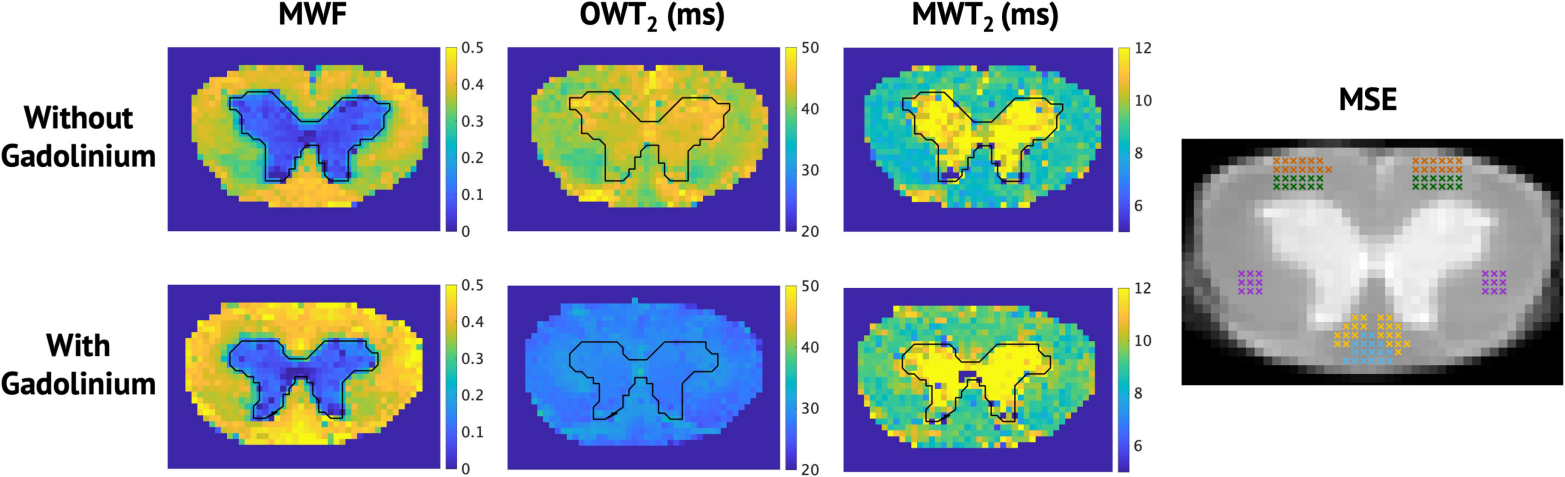
Parameter maps for myelin water fraction, other water T_2_, and myelin water T_2_, with and without gadolinium The black line outlines the border between white and gray matter. On the far right, the first echo image is displayed with five ROIs that were selected for further analysis.

Mean and standard deviation values were calculated across manually selected white and gray matter regions of interest (ROIs), respectively, in datasets both with and without Gd. Values were plotted, and statistically significant differences were evaluated with a rank sum test and deemed significant for *p* < 0.002 with an applied Bonferroni correction for 30 comparisons. Additionally, relationships between qMT and MET_2_‐derived metrics, and the diffusion MRI‐derived axon diameter surrogate, ∆D_⊥_ [14, 16], for previously established ROIs [14] (displayed in Figure 1) were evaluated via linear regression across spinal cords and deemed statistically significant for *p* < 0.005 with an applied Bonferroni correction for 10 comparisons.

## 3 Results

### 3.1 MSE‐Derived Metrics

**FIGURE 2:**
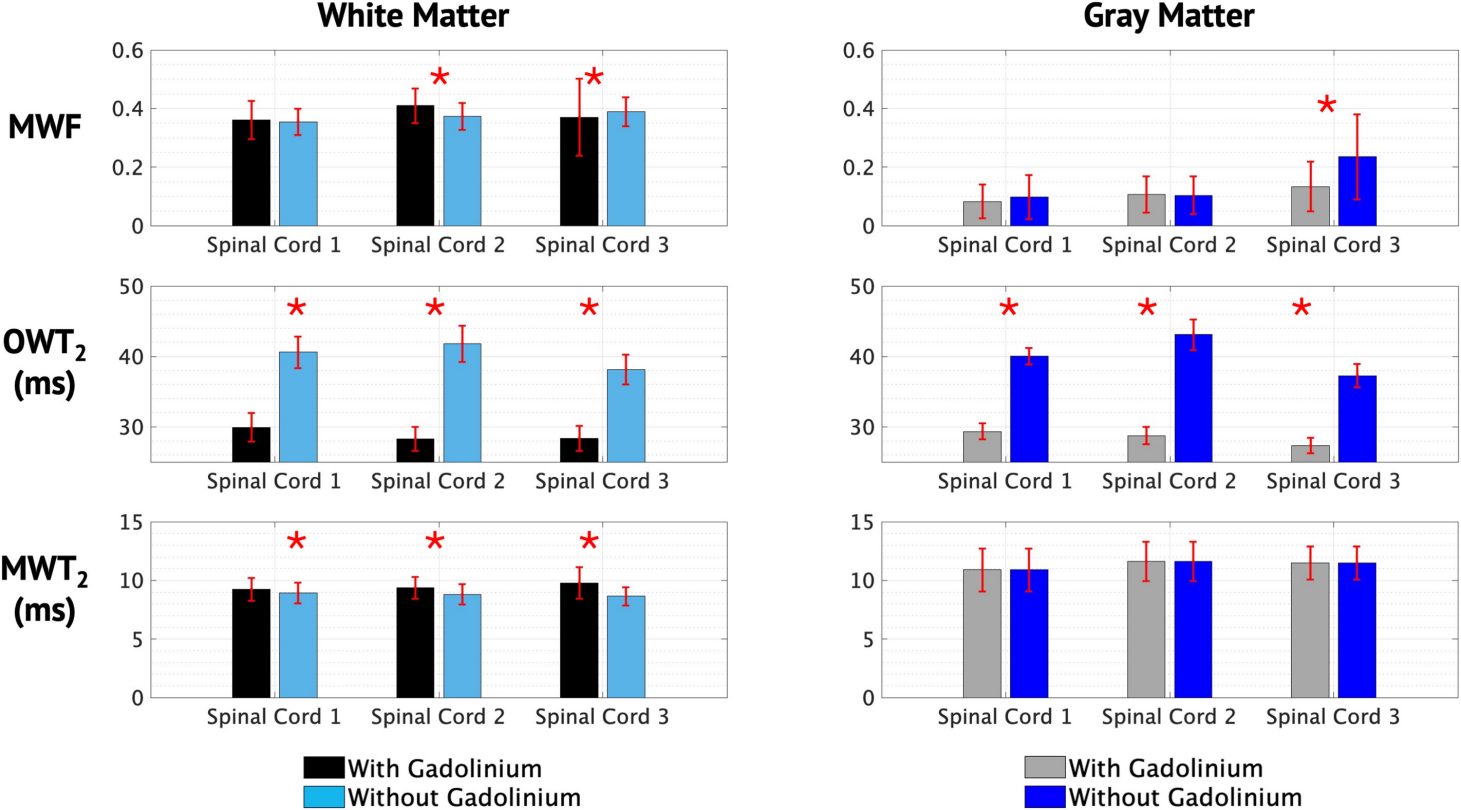
Mean MET_2_‐derived parameters across the white and gray matter, for three different spinal cords with and without gadolinium. Error bars represent standard deviations, and the asterisks represent statistically significant differences between the with and without gadolinium means.

Representative quantitative parameter maps from a spinal cord are displayed in Figure 1. As expected, there were clear changes in OWT_2_ with and without Gd, while MWF and MWT_2_ showed more subtle changes. Figure 2 displays bar graphs of mean parameters ± standard deviation over both white and gray matter for all three spinal cords with and without Gd. MWF with and without Gd was similar in the white matter of all three spinal cords. A significant difference was found in spinal cords 2 and 3 (*p* < < 0.001), though the difference was in opposite directions, indicating that scan–rescan variability was larger than biases introduced by the presence of Gd. Similarly, changes in MWT_2_ with and without Gd was subtle but significant for all spinal cords (*p* ≪ 0.001). As expected, OWT_2_ is significantly longer in both white and gray matter without Gd (*p* < < 0.001). MET_2_‐derived metrics averaged across spinal cords for white and gray matter with and without Gd are summarized in Table 1.

**TABLE 1:** qMRI parameters averaged across spinal cords (*N* = 3) ± standard deviations for both white matter (WM) and gray matter (GM) with and without gadolinium.

	MWF	OWT_2_ (ms)	MWT_2_ (ms)	BPF	T_1,f_ (ms)	*k*_*mf*_ (s^−1^)
WM_Gd_	0.38 s3 ± 0.091	28.8 ± 1.95	9.45 ± 1.10	0.224 ± 0.019	225 ± 12.2	14.7 ± 1.36
WM_noGd_	0.371 ± 0.048	40.5 ± 2.78	8.82 ± 0.848	0.236 ± 0.020	1051 ± 102	13.0 ± 0.892
GM_Gd_	0.104 ± 0.068	28.6 ± 1.37	11.4 ± 1.70	0.192 ± 0.013	221 ± 9.17	16.7 ± 1.13
GM_noGd_	0.129 ± 0.104	41.1 ± 3.01	11.4 ± 1.70	0.203 ± 0.021	1135 ± 114	14.3 ± 1.07

### 3.2 SIR‐Derived Metrics

**FIGURE 3:**
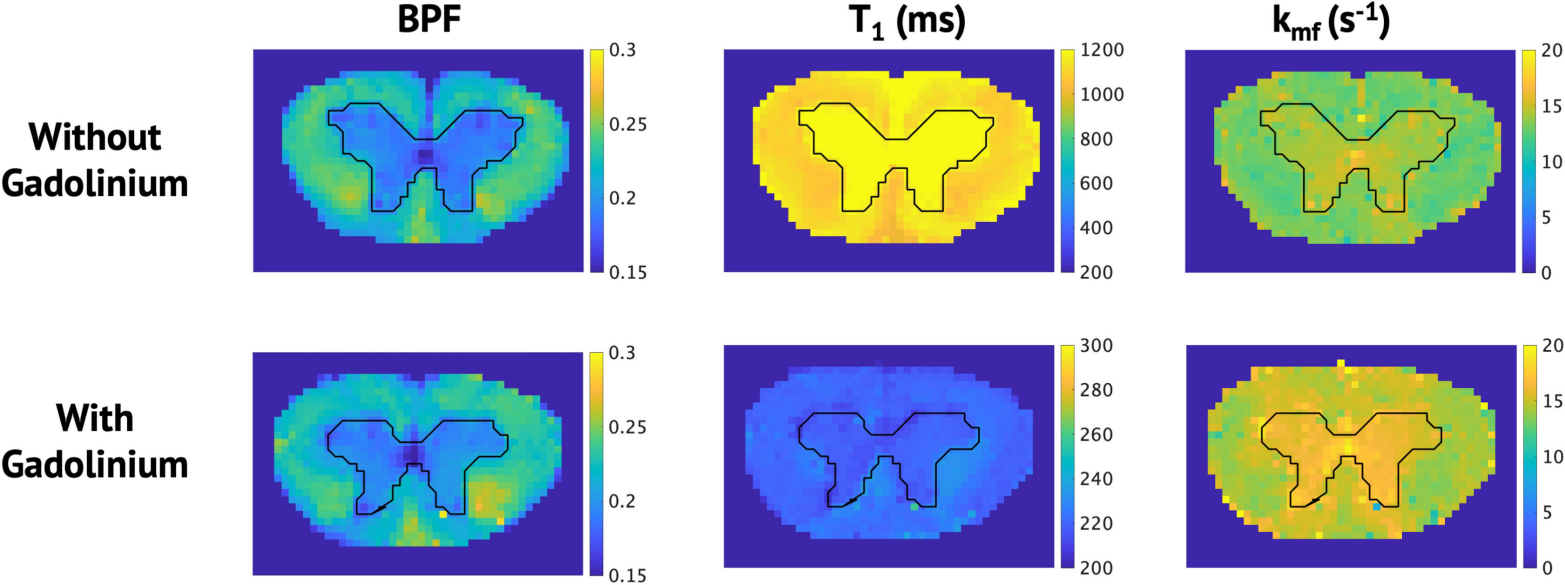
Parameter maps for bound pool fraction and T1 of the free proton pool, with and without gadolinium. The black line distinguishes white and gray matter.

**FIGURE 4:**
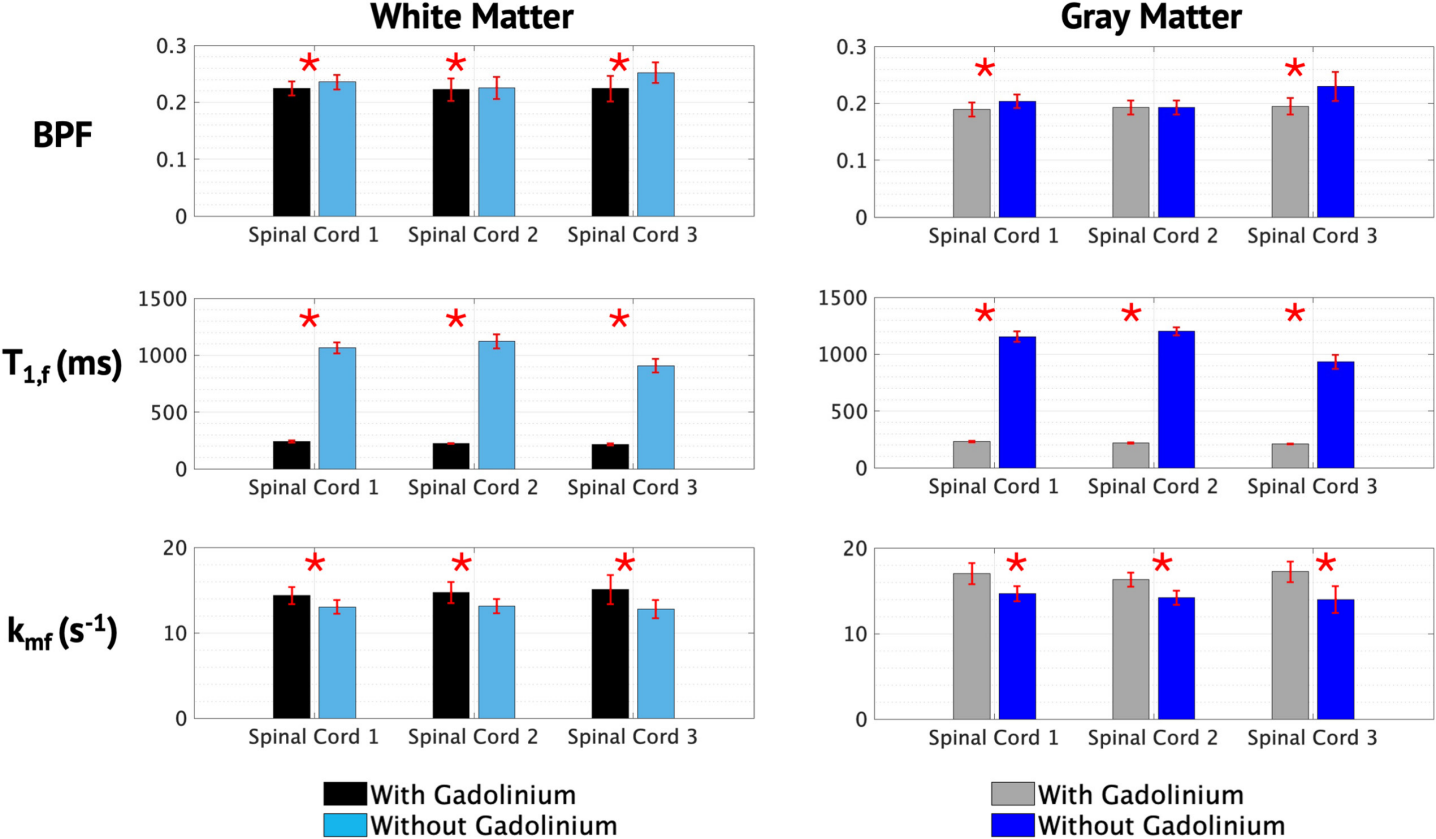
Mean qMT‐derived parameters across the white and gray matter, for three different spinal cords with and without gadolinium. Error bars represent standard deviations, and the asterisks represent statistically significant differences between the with and without gadolinium means.

SIR‐derived parameter maps are shown in Figure 3. Similar to OWT_2_, T_1f_ shows global and clear differences between maps acquired with and without Gd, while BPF shows very little change. Figure 4 displays a quantitative evaluation of the effect of Gd on BPF and T_1f_. Similar to MWF, changes in BPF in white matter with and without Gd were subtle but statistically significant (*p* < 0.001). Similar to OWT_2_, T_1f_ is significantly longer in tissues without Gd (*p* < 0.001). Although not commonly reported, *k*_*mf*_ is also presented for completeness. In Figure 3, *k*_*mf*_ appears higher in the spinal cord with Gd. This is supported quantitatively with significant differences across white and gray matter (Figure 4). SIR‐derived metrics averaged across spinal cords for white and gray matter are included in Table 1.

### 3.3 Myelin Metrics and Axon Diameter Surrogate

**FIGURE 5:**
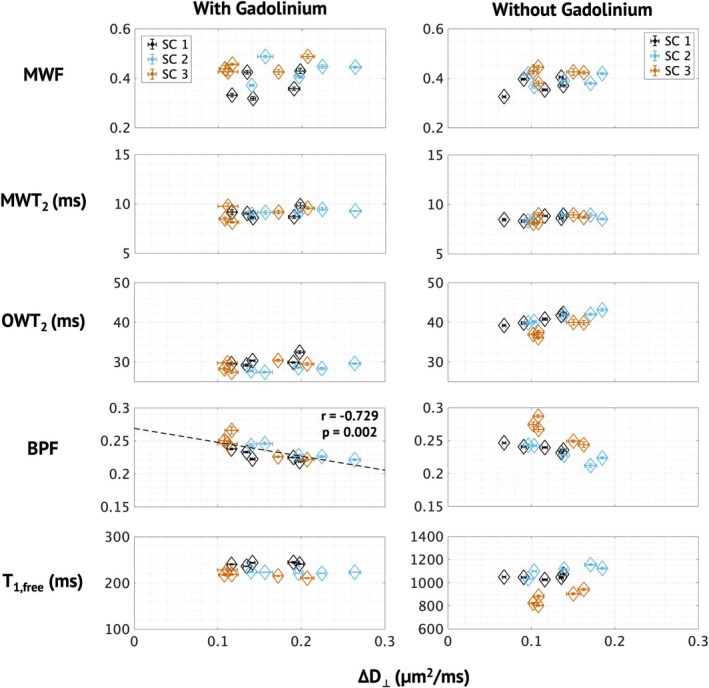
Parameters from each spinal cord averaged across ROIs selected in Alderson et al. [14] and plotted against the recently validated axon diameter surrogate, ∆D_⟂_, with and without gadolinium.

Figure 5 displays the relationship between relaxometry‐derived metrics and the recently proposed axon diameter surrogate, ∆D_⊥_^14,16,^for five ROIs and all three spinal cords. BPF was found to have a significantly negative correlation with ∆D_⊥_ with Gd (*r* = −0.729) and a negative but insignificant trend without Gd. OWT_2_ was found to have no relationship with ∆D_⊥_ when Gd was present in the tissue, but a positive, though insignificant, trend with ∆D_⊥_ without Gd (*p* = 0.02). Though T_1f_ was not found to have a relationship with ∆D_⊥_ with or without Gd, similar trends to OWT_2_ can be observed. That is, without Gd, there appears to be increasing T_1f_ with increasing ∆D_⊥_, and with Gd, this trend is flattened. Lastly, neither MWT_2_ nor MWF were found to have a significant relationship with ∆D_⊥_ with or without Gd.

## 4 Discussion

This work evaluates the impact of using Gd in ex vivo spinal cords on myelin MRI metrics, as well as the effect on the relationship between those metrics and a surrogate measure of axon diameter, ∆D_⊥_. As expected, Gd primarily impacted OWT_2_ and T_1f_ in the tissues evaluated here. Specifically, they were both significantly shorter with Gd than without, as expected due to the relaxivity of Gd‐based contrast agents. This effect was observed globally in both white and gray matter. MWF and BPF were both shown to have subtle but statistically significant differences between acquisitions acquired with and without Gd, likely due to scan re‐scan variability. Furthermore, the average percent difference across these values between with and without Gd means was 6% (supporting information). Depending on the application, this magnitude may not be of concern. A study looking at intra‐myelinic edema found ~13%–75% differences in PSR (a metric similar to BPF) and MWF between disease and control rat spinal cords [17]. Another study observed ~16%–80% differences between MWF and BPF in the rat corpus callosum between control and knock‐out rats experiencing demyelination [18].

The T_1_ and T_2_ values reported here are not derived from a monoexponential fit, as they are typically reported. Instead, these metrics are derived from multi‐compartment models and are assumed to represent specific pools of protons. Still, the relaxivities of Gd calculated from the Gd and non‐Gd data reported here (Table 2) are comparable with values reported in the macaque [5], mouse brain [4], and water [4]. These literature values range from 2.1 to 3.88 mM^−1^ s^−1^ for r_1,Gd_ and 5.43 to 13.8 mM^−1^ s^−1^ for r_2,Gd_ at 14 T, with the low end corresponding to Gd in water. Furthermore, West presents relaxivities in ex vivo rat brains of r_2,Gd_ = 2.81 (s^−1^/mM) and r_1,Gd_ = 15.96 (s^−1^/mM) respectively for a Gd concentration of 0.5 mM [19].

**TABLE 2:** Relaxivities of gadolinium (ProHance) calculated from the equations displayed in the top row of the table, based on the experimental values (R_1f_, R_1f0_, OWR_2_, and OWR_2,0_).

	R_1f_ = R_1f,0_ + r_1,Gd_[Gd]		OWR_2_ = OWR_2,0_ + r_2,Gd_[Gd]	
	R_1f,0_ (s^−1^)	r_1,Gd_ (s^−1^ mM^−1^)	OWR_2,0_ (s^−1^)	r_2,Gd_ (s^−1^ mM^−1^)
WM	0.951	3.49	24.7	10.0
GM	0.881	3.64	24.3	10.67

While there are currently no T_1f_ values available in the literature for the ex vivo ferret spinal cord, those measured in rat spinal cord are comparable with the values found without Gd here. Specifically, Harkins et al. published T_1f_ values in the ex vivo rat spinal cord at 9.4T [20]. These values were estimated without Gd and found to be 824–941 ms for different white matter regions, which is comparable with the mean value of 1051 ms reported here. Similarly, OWT_2_ and MWT_2_ values without Gd in the ex vivo rat spinal cord (43.9–60.9 ms and 10.8–14 ms, respectively) are comparable with the values reported here (40.5 and 8.82 ms, respectively). Additionally, qualitative examination of T_1f_ maps did not indicate that Gd was partially or nonuniformly washed out of the tissues.

The values of *k*_*mf*_ reported here are comparable with those in other literature. One study reported values of *k*_*mf*_ = 13.1 ± 2.9 and 20.8 ± 6.5 s^−1^ in the white and gray matter respectively in vivo rat brains [21]. Another study reported *k*_*mf*_ values of 15–20 s^−1^ across various corpus callosum regions in ex vivo rat brains [22]. The difference in *k*_*mf*_ measured with and without Gd was unexpected, as Gd is not thought to affect rates of magnetization transfer between proton pools. It is possible that the difference in the range of experimental TI—which differed due to the shortening of T_1_ in the presence of Gd—could cause some bias in *k*_*mf*_. It is also possible the difference is caused by oversimplifying assumptions of the two‐compartment model used for qMT analysis. Further work is required to fully understand the mechanisms underlying any effect of Gd on *k*_*mf*_.

**FIGURE 6:**
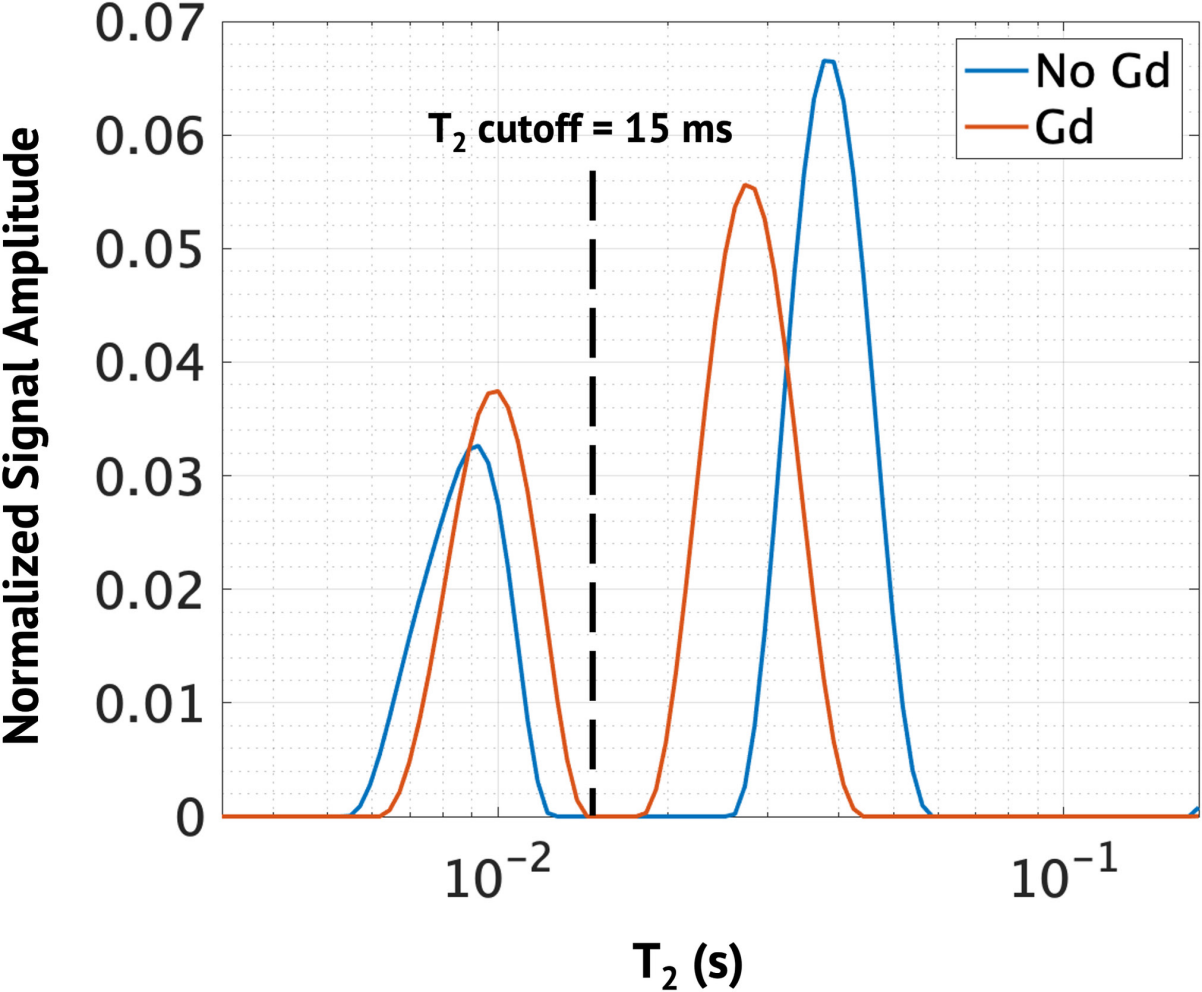
T_2_ spectra from representative voxels with and without gadolinium. The dashed line represents the cutoff T_2_ value used for the calculation of MWF.

As described in the introduction, MET_2_ analysis assumes that T_2_ contributions arise from anatomically distinct compartments that undergo slow exchange. In the central nervous system, this manifests in two observable compartments in the T_2_ spectra: myelin water and a combined extra‐ and intra‐axonal compartment. Anything that impacts the relaxation of one of these compartments has the potential to impact MWF. For example, a shift in the OWT_2_ closer to MWT_2_ could make it more difficult to distinguish the two compartments. Figure 6 shows example spectra from datasets with and without Gd. In the Gd dataset, the OWT_2_ and MWT_2_ peaks have shifted closer together compared with the spectra without Gd. A slight but noticeable shift in the relative size of the peaks can also be observed, which results in a higher MWF (0.344 to 0.392). However, the ROI values displayed in Figure 5 show comparable ranges of MWF with and without Gd, and the trend with the axon diameter surrogate, ∆D_⊥_, is not impacted.

In contrast to MET_2_ analysis, qMT analysis is based on the exchange of magnetization between distinct molecular pools of protons: those that are “free” and those that are “bound” to macromolecules [13, 23]. This exchange results in an observed T_1_ recovery that appears biexponential [13, 23], and MT metrics are estimated from the magnitude and rate of the short T_1_ component. Therefore, Gd could bias qMT‐derived parameters if the T_1_ of free water was impacted in a way that interfered with the exchange process between these two pools that are assumed to be homogeneously mixed. The average mean squared errors across both the Gd spinal cords and no Gd spinal cords are very low (1.40 × 10^−5^ and 5.53 × 10^−5^, respectively) and indicate that the two‐pool qMT model fits both the Gd and without Gd datasets well. Similar to MWF, the ROI analysis reveals that the range of BPF is comparable with and without Gd, and its relationship to ∆D_⊥_ is not impacted.

No definitive claim can be made based on the data presented here regarding the distribution of Gd in ex vivo tissue; however, it is worth noting what may be expected in the MET_2_ and qMT results if Gd was unevenly distributed amongst the compartments of interest. One might expect a third relaxation component in qMT and MET_2_ datasets. For MET_2_, this may manifest as either a broader free water peak or the introduction of a third T_2_ component in the T_2_ spectra. For qMT, the emergence of a third T_1_ component could increase the overall error in the qMT fitted dataset. While these changes were not observed in the data presented here, future experiments focused on investigating the Gd distribution may use compartment models to do so. Peripheral nerve could also be used to further investigate the distribution of Gd in ex vivo tissue. Though there are only two observable peaks in the T_2_ spectra of the central nervous system, three peaks are observed in peripheral nerve. That could allow for a more direct evaluation of the impact of Gd on intra‐axonal, extra‐axonal, and myelin compartments. Nonetheless, the focus of the work presented here was on investigating the impacts of Gd on commonly used qMRI white matter methods.

Additionally, future work may entail disentangling the impacts of fixation versus Gd on the qMRI parameters estimated here. Many authors have investigated the impact of fixation on relaxation [24], diffusion [24, 25], and myelin [26] MRI parameters; however, experiments evaluating the effects of both Gd and fixative effects could be of great value. It is also important to note that the different types of fixatives may have different impacts on tissue microstructure.

Lastly, the methodology of this work has a few limitations. First, samples were first soaked in PBS + Gd before being washed with only PBS, as opposed to starting without Gd and adding it after initial scans. This could bias the relaxation metrics if any Gd remains after washing out. This study also used only a small number of samples (*N* = 3). However, the results remain consistent across the samples evaluated here—the presence of Gd primarily impacts the relaxation and has a significant but negligible impact on the myelin content measures. Furthermore, these results are obtained from the evaluation of simple tissues. Spinal cords are known to have high alignment of white matter axons and very little dispersion. It would be worthwhile to repeat these investigations in the brain to evaluate more complex anatomy. Specifically, recent literature suggests that at least some of the metrics evaluated here may be dependent on axon orientation in the brain [27]; thus, including orientation and Gd in one study may be of future consideration.

## 5 Conclusion

In the ex vivo spinal cords evaluated in this work, Gd primarily impacted T_1f_ derived from qMT analysis of SIR data and OWT_2_ derived from MET_2_ analysis of MSE data. Significant differences were also found for metrics reporting on myelin content; however, the magnitude of these differences is likely small compared with those observed in pathology or in some cases even scan‐rescan variations.

## Author Contributions

Mark Does and Kevin Harkins contributed through supervision, discussions regarding experimental design and interpreting the results, funding acquisition, and provided editorial revisions to the manuscript. Hannah Alderson conducted the experiments, contributed to the conception and design of the experiments, and wrote the manuscript.

## Conflicts of Interest

The authors declare no conflicts of interest.

## Supporting information




**TABLE S1:** MET_2_‐derived metrics for each spinal cord, with and without gadolinium. The *p*‐values from ranksum comparisons and percent changes due to Gd are provided.

**TABLE S2:** SIR‐derived metrics for each spinal cord, with and without gadolinium. The *p*‐values from ranksum comparisons and percent changes due to Gd are provided.

## Data Availability

The data that support the findings of this study are available on request from the corresponding author.
