# Supplementary material for: The Impact of Gadolinium on Quantitative Myelin Metrics in Ex Vivo Spinal Cord
Source: NMR Biomed. 2026 Mar 22;39(5):e70275. doi: 10.1002/nbm.70275 (PMC13006719; doi:10.1002/nbm.70275)
Supplement: Supplementary file 1 — TABLE S1: MET2‐derived metrics for each spinal cord, with and without gadolinium. The p‐values from ranksum comparisons and percent changes due to Gd are provided. [file NBM-39-e70275-s001.pptx]

## Slide 1
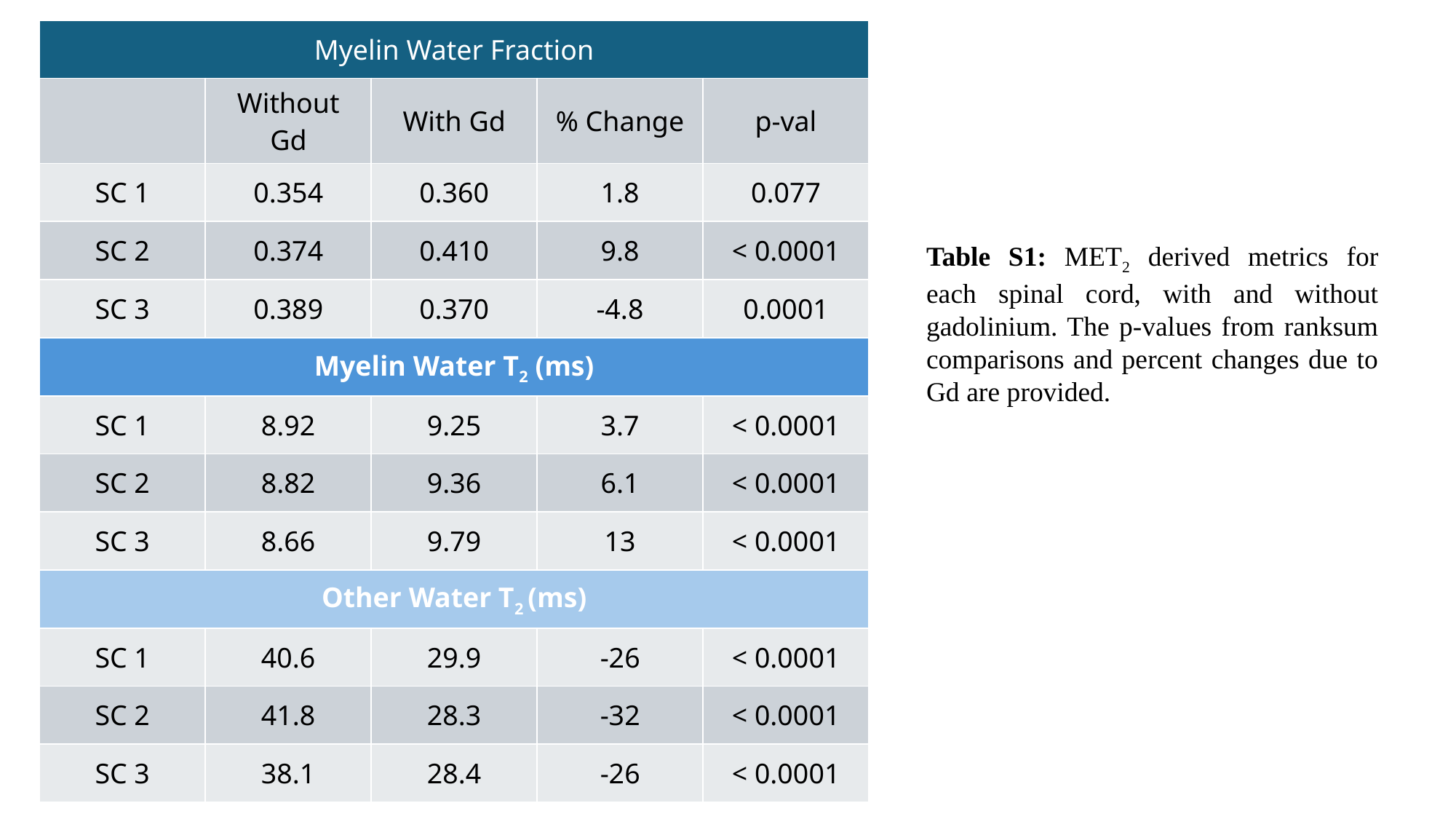

| Myelin Water Fraction | | | | |
| --- | --- | --- | --- | --- |
| | Without Gd | With Gd | % Change | p-val |
| SC 1 | 0.354 | 0.360 | 1.8 | 0.077 |
| SC 2 | 0.374 | 0.410 | 9.8 | < 0.0001 |
| SC 3 | 0.389 | 0.370 | -4.8 | 0.0001 |
| Myelin Water T2 (ms) | | | | |
| SC 1 | 8.92 | 9.25 | 3.7 | < 0.0001 |
| SC 2 | 8.82 | 9.36 | 6.1 | < 0.0001 |
| SC 3 | 8.66 | 9.79 | 13 | < 0.0001 |
| Other Water T2 (ms) | | | | |
| SC 1 | 40.6 | 29.9 | -26 | < 0.0001 |
| SC 2 | 41.8 | 28.3 | -32 | < 0.0001 |
| SC 3 | 38.1 | 28.4 | -26 | < 0.0001 |
Table S1: MET2 derived metrics for each spinal cord, with and without gadolinium. The p-values from ranksum comparisons and percent changes due to Gd are provided.
