# Supplementary material for: The Impact of Gadolinium on Quantitative Myelin Metrics in Ex Vivo Spinal Cord
Source: NMR Biomed. 2026 Mar 22;39(5):e70275. doi: 10.1002/nbm.70275 (PMC13006719; doi:10.1002/nbm.70275)
Supplement: Supplementary file 2 — TABLE S2: SIR‐derived metrics for each spinal cord, with and without gadolinium. The p‐values from ranksum comparisons and percent changes due to Gd are provided. [file NBM-39-e70275-s002.pptx]

## Slide 1
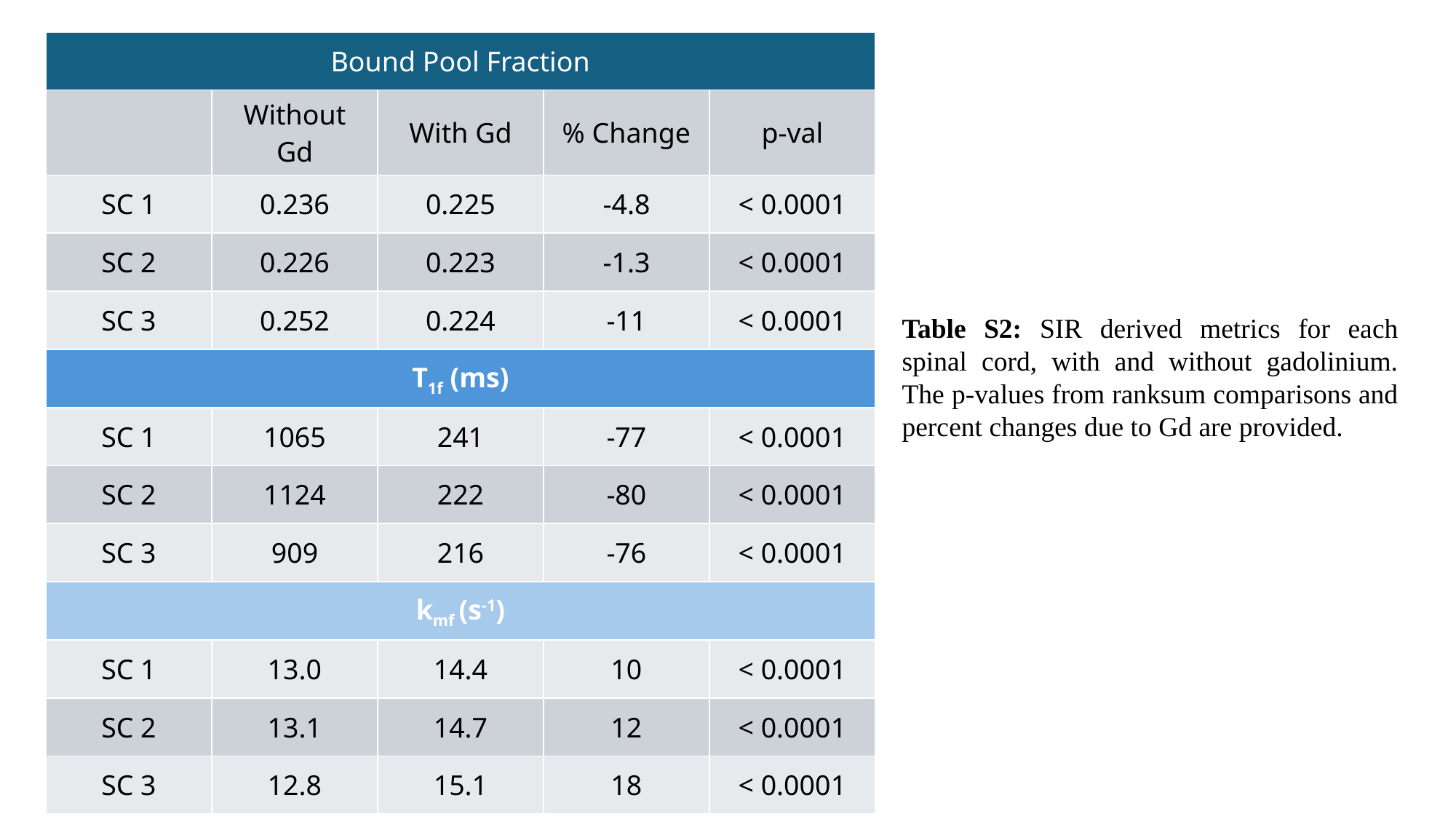

| Bound Pool Fraction | | | | |
| --- | --- | --- | --- | --- |
| | Without Gd | With Gd | % Change | p-val |
| SC 1 | 0.236 | 0.225 | -4.8 | < 0.0001 |
| SC 2 | 0.226 | 0.223 | -1.3 | < 0.0001 |
| SC 3 | 0.252 | 0.224 | -11 | < 0.0001 |
| T1f (ms) | | | | |
| SC 1 | 1065 | 241 | -77 | < 0.0001 |
| SC 2 | 1124 | 222 | -80 | < 0.0001 |
| SC 3 | 909 | 216 | -76 | < 0.0001 |
| kmf (s-1) | | | | |
| SC 1 | 13.0 | 14.4 | 10 | < 0.0001 |
| SC 2 | 13.1 | 14.7 | 12 | < 0.0001 |
| SC 3 | 12.8 | 15.1 | 18 | < 0.0001 |
Table S2: SIR derived metrics for each spinal cord, with and without gadolinium. The p-values from ranksum comparisons and percent changes due to Gd are provided.
